# The Diabetes Self-Management Questionnaire (DSMQ): development and evaluation of an instrument to assess diabetes self-care activities associated with glycaemic control

**DOI:** 10.1186/1477-7525-11-138

**Published:** 2013-08-13

**Authors:** Andreas Schmitt, Annika Gahr, Norbert Hermanns, Bernhard Kulzer, Jörg Huber, Thomas Haak

**Affiliations:** 1 Research Institute of the Diabetes Academy Mergentheim (FIDAM), German Diabetes Center Mergentheim, Theodor-Klotzbücher-Str. 12, D-97980 Bad Mergentheim, Germany; 2 The University of Northampton, Boughton Green Rd, Northampton NN2 7AL, UK

**Keywords:** Diabetes care, Self-management, Self-care behaviour, Metabolic control, HbA_1c_, Hyperglycaemia, Measurement, Assessment, Psychometric instrument

## Abstract

**Background:**

Though several questionnaires on self-care and regimen adherence have been introduced, the evaluations do not always report consistent and substantial correlations with measures of glycaemic control. Small ability to explain variance in HbA_1c_ constitutes a significant limitation of an instrument’s use for scientific purposes as well as clinical practice. In order to assess self-care activities which can predict glycaemic control, the Diabetes Self-Management Questionnaire (DSMQ) was designed.

**Methods:**

A 16 item questionnaire to assess self-care activities associated with glycaemic control was developed, based on theoretical considerations and a process of empirical improvements. Four subscales, ‘Glucose Management’ (GM), ‘Dietary Control’ (DC), ‘Physical Activity’ (PA), and ‘Health-Care Use’ (HU), as well as a ‘Sum Scale’ (SS) as a global measure of self-care were derived. To evaluate its psychometric quality, 261 patients with type 1 or 2 diabetes were assessed with the DSMQ and an established analogous scale, the Summary of Diabetes Self-Care Activities Measure (SDSCA). The DSMQ’s item and scale characteristics as well as factorial and convergent validity were analysed, and its convergence with HbA_1c_ was compared to the SDSCA.

**Results:**

The items showed appropriate characteristics (mean item-total-correlation: 0.46 ± 0.12; mean correlation with HbA_1c_: -0.23 ± 0.09). Overall internal consistency (Cronbach’s alpha) was good (0.84), consistencies of the subscales were acceptable (GM: 0.77; DC: 0.77; PA: 0.76; HU: 0.60). Principal component analysis indicated a four factor structure and confirmed the designed scale structure. Confirmatory factor analysis indicated appropriate fit of the four factor model. The DSMQ scales showed significant convergent correlations with their parallel SDSCA scales (GM: 0.57; DC: 0.52; PA: 0.58; HU: n/a; SS: 0.57) and HbA_1c_ (GM: -0.39; DC: -0.30; PA: -0.15; HU: -0.22; SS: -0.40). All correlations with HbA_1c_ were significantly stronger than those obtained with the SDSCA.

**Conclusions:**

This study provides preliminary evidence that the DSMQ is a reliable and valid instrument and enables an efficient assessment of self-care behaviours associated with glycaemic control. The questionnaire should be valuable for scientific analyses as well as clinical use in both type 1 and type 2 diabetes patients.

## Background

Hyperglycaemia is a major predictor of the development of diabetes late complications, and improving glycaemic control has been shown to prevent microvascular as well as macrovascular events (the latter at least in type 1 diabetes) [1-3]. Although a number of internal and external factors contribute to the level of blood glucose [4], it is widely accepted that good self-care protects against complications in both type 1 and type 2 diabetes and that the patient must actively manage the disease’s requirements in order to achieve optimal blood glucose outcomes [1,5,6].

It has often been suggested that important psychosocial factors such as depression and emotional distress can interfere with self-care behaviours and therefore negatively impact glycaemic control [7,8]. Consequently, numerous studies have concentrated on negative emotional conditions and actually found associations with both reduced self-care activities [9,10] and elevated HbA_1c_ values [11-15]. However, research has yielded only limited insight into the suggested behavioural mechanisms between negative affect and hyperglycaemia, and this is to be explained at least partially by methodological problems of construct assessment.

A promising way to study such mechanisms is to utilise multiple regression or structural equation modeling and analyse the putative mediation of the relationship between an affective condition and HbA_1c_ by self-care. However, the applicability of this method and the conclusiveness of its results strongly depend on the self-care assessment’s ability to explain variance in the criterion variable [16]. If the measuring instrument is not sufficiently associated with HbA_1c_, the putative mediation may actually not be observed. For example, this may have been the case with the analysis by Lustman et al. [17], who found an association between depression and hyperglycaemia but no mediation of the association by self-care behaviour.

Taken as a whole, weak associations with glycaemic outcomes [18-20] or the omission of reporting the critical data [21,22] can be frequently found among evaluations of eligible questionnaires, but there are also further obstacles. A recent review of psychometric tools identified a total of five questionnaires which assess self-management, but only one fully met the reviewers’ appraisal criteria [23].

That one questionnaire, which satisfied the reviewers’ expectations, was the Summary of Diabetes Self-Care Activities Measure (SDSCA), which is probably the most popular and most frequently used instrument in its regard. It has been evaluated in numerous studies, shown appropriate psychometric qualities and been translated into many languages. However, the authors stated that the questionnaire was not conceptualized to be closely linked to glycated haemoglobin, and consequently, its initial evaluation did not find any significant associations between its scales and HbA_1c_[19]. Later studies have confirmed this lack of correlation, and to our knowledge no studies presenting moderate or strong correlations between the SDSCA and glycated haemoglobin have been reported [22,24-28].

While a valid assessment of diabetes self-care does not necessarily need to correlate with glycaemic outcome, a weak association between an instrument and HbA_1c_ nevertheless constitutes a major limitation for its use in research and also for practitioners interested in helping patients to improve or maintain good glycaemic control.

In order to facilitate the collection of appropriate data, the Diabetes Self-Management Questionnaire (DSMQ) was developed. The questionnaire was designed to assess self-care behaviours which can be related to the measure of HbA_1c_, so that the data are suitable for mediational analyses. A second objective was to construct a brief instrument suitable for studies involving a multitude of data collection instruments including clinical trials. This article describes the DSMQ’s development and presents its first psychometric evaluation.

## Methods

Two studies were conducted at the German Diabetes Center Mergentheim (GDCM), a tertiary referral centre for diabetes (Patients may be referred to the centre for different reasons. Providing intensive diabetes education, treating substantial problems of diabetes control, or performing major changes regarding a patient’s therapy may be typical reasons for referral. The average time of the stay is about 10 days.). Study 1 evaluated an initial set of 37 items on 110 in-patients, resulting in a final questionnaire containing 16 items. Study 2 assessed the psychometric properties of this 16-item scale on 261 in-patients.

Study participation was limited to patients with type 1 or 2 diabetes, adult age, sufficient German language skills, and providing informed consent. In-patients who met inclusion criteria were informed about the possibility to participate in a cross-sectional study of questionnaire evaluation. Patients who consented were assessed with the DSMQ and the SDSCA. Additionally, demographic and diabetes-specific characteristics were gained from the electronic patient records (sex, age, BMI, diabetes type, diabetes duration, type of diabetes treatment, late complication status, and current HbA_1c_). Both study samples reflected the typical clinic population composition, which mainly comprises of type 1 and type 2 diabetes in approximately equal percentages as well.

Data collection was carried out during a supplementary cross-sectional survey of the DIAMOS study (‘Strengthening Diabetes Motivation’) (Identifier: NCT01009138), approved by the Ethics Committee of the State Medical Chamber of Baden-Wuerttemberg (file number 2009-034-f). Written informed consent was obtained before participation.

### Instruments and measures

#### Development of the Diabetes Self-Management Questionnaire (DSMQ)

The DSMQ was developed at the Research Institute of the Diabetes Academy Mergentheim. It is the first German instrument targeting diabetes self-care, and was designed to assess behaviours associated with metabolic control within common treatment regimens for type 1 and type 2 diabetes in adult patients.

Initially, 37 items were generated with contents which, in view of the literature, were regarded as confirmed or promising predictors of glycaemic control. In this regard, the accuracy of medication intake and diet adjustment were regarded as important predictors in both type 1 and 2 diabetes. Poor adherence to insulin as well as oral medical regimens has been consistently associated with hyperglycaemia [29-33], and the change to a diet with a lower glycaemic index has shown the potential of improving glycaemic control regardless of diabetes type [34,35].

Another content of interest is self-monitoring of blood glucose (SMBG) as its impact on glycaemic control is well-established in type 1 as well as type 2 diabetes with insulin treatment [36,37]. Although there is uncertainty and debate about its benefit in insulin-naive patients [38], several studies suggest SMBG can be also advantageous in those [39-41], particularly when the feedback leads to relevant action [42]. Furthermore, two recent publications comparably concluded that SMBG can very well be an effective means of glycaemic control in insulin-naive patients if used in a structured and knowledgeable way [43,44].

Physical exercise as a means of metabolic control is commonly used in type 2 diabetes, and its effectiveness is well established [45,46]. Nevertheless, a recent meta-analysis found that exercise is also effective in improving HbA_1c_ levels in type 1 diabetes [47]. Therefore, physical activity (particularly with regard to diabetes treatment) was regarded as appropriate item content.

Finally, some items were designed to assess the patient’s adherence to (vs. avoidance of) appointments with health-care professionals, which, compared to previous questionnaires, is a somewhat new aspect. However, a higher frequency of primary care contacts is associated with a better glycaemic outcome [48], and the commonly motivating effect of feedback on HbA_1c_ is one putative explanation of this finding [49]. Furthermore, appointment adherence was found to predict glycaemic control independently of visit frequency [50,51]. Finally, appointment adherence seems to be reduced in depressed diabetes patients [9]. Therefore, this aspect should not be missed out when studying psychosocial predictors of diabetes control.

The final set of items tested in study 1 comprised of the following contents: Regularity of medication intake (4 items), diabetes-related aspects of diet (e. g. frequent consumption of foods complicating glycaemic control, adherence to dietary recommendations, alcohol consumption; 8 items), regularity of self-monitoring of blood glucose (4 items), regularity of physical activity (5 items), appointment adherence (4 items), several specific self-care activities, e. g. carriage of needed therapy devices, adequate treatment of hypoglycaemic/ hyperglycaemic episodes, record of blood glucose levels (5 items), and overall judgements of the adequacy of self-care (7 items). The items then were reviewed by a team of five psychologists, three diabetologists, and a sample of 15 diabetes patients, leading to the final item formulation.

All items were formulated as behavioural descriptions taking the first person view. Respondents are asked to rate the extent to which each statement applies to the personal self-management with regard to the previous eight weeks. The time frame was chosen in view of the specific time-dependence of HbA_1c_ values [52,53], as recommended by Johnson [4]. The rating scale was designed as a four-point Likert scale (in order to avoid a neutral response option and force a specific response) with the response options ‘applies to me very much’ (three points), ‘applies to me to a considerable degree’ (two points), ‘applies to me to some degree’ (one point), and ‘does not apply to me’ (zero points). The responses were converted such that higher scores are indicative of more effective self-care. To enable individual adjustment in items which assess aspects of SMBG or medical treatment, boxes offering to tick ‘is not required as a part of my treatment’ were added.

Analysis of responses as part of study 1 led to the identification of 16 items which formed the final scale for full psychometric assessment. Seven of these items are formulated positively and nine inversely with regard to what is considered effective self-care. The questionnaire allows the summation to a ‘Sum Scale’ score as well as estimation of four subscale scores. In view of their contents, the subscales were labelled ‘Glucose Management’ (items 1, 4, 6, 10, 12), ‘Dietary Control’ (items 2, 5, 9, 13), ‘Physical Activity’ (items 8, 11, 15), and ‘Health-Care Use’ (items 3, 7, 14). One item (16) requests an overall rating of self-care and is to be included in the ‘Sum Scale’ only. The full questionnaire is displayed in Table 1.

**Table 1:** Diabetes Self-Management Questionnaire (DSMQ)

**The following statements describe self-care activities related to your diabetes. Thinking about your self-care over the last 8 weeks, please specify the extent to which each statement applies to you.**		**Applies to me very much**	**Applies to me to a consider-able degree**	**Applies to me to some degree**	**Does not apply to me**
1.	I check my blood sugar levels with care and attention. ☐ *Blood sugar measurement is not required as a part of my treatment.*	☐3	☐2	☐1	☐0
2.	The food I choose to eat makes it easy to achieve optimal blood sugar levels.	☐3	☐2	☐1	☐0
3.	I keep all doctors’ appointments recommended for my diabetes treatment.	☐3	☐2	☐1	☐0
4.	I take my diabetes medication (e. g. insulin, tablets) as prescribed. ☐ *Diabetes medication / insulin is not required as a part of my treatment.*	☐3	☐2	☐1	☐0
5.	Occasionally I eat lots of sweets or other foods rich in carbohydrates.	☐3	☐2	☐1	☐0
6.	I record my blood sugar levels regularly (or analyse the value chart with my blood glucose meter). ☐ *Blood sugar measurement is not required as a part of my treatment.*	☐3	☐2	☐1	☐0
7.	I tend to avoid diabetes-related doctors’ appointments.	☐3	☐2	☐1	☐0
8.	I do regular physical activity to achieve optimal blood sugar levels.	☐3	☐2	☐1	☐0
9.	I strictly follow the dietary recommendations given by my doctor or diabetes specialist.	☐3	☐2	☐1	☐0
10.	I do not check my blood sugar levels frequently enough as would be required for achieving good blood glucose control. ☐ *Blood sugar measurement is not required as a part of my treatment.*	☐3	☐2	☐1	☐0
11.	I avoid physical activity, although it would improve my diabetes.	☐3	☐2	☐1	☐0
12.	I tend to forget to take or skip my diabetes medication (e. g. insulin, tablets). ☐ *Diabetes medication / insulin is not required as a part of my treatment.*	☐3	☐2	☐1	☐0
13.	Sometimes I have real ‘food binges’ (not triggered by hypoglycaemia).	☐3	☐2	☐1	☐0
14.	Regarding my diabetes care, I should see my medical practitioner(s) more often.	☐3	☐2	☐1	☐0
15.	I tend to skip planned physical activity.	☐3	☐2	☐1	☐0
16.	My diabetes self-care is poor.	☐3	☐2	☐1	☐0

Scoring of the questionnaire involved reversing negatively worded items such that higher values are indicative of more effective self-care. Scale scores were calculated as sums of item scores and then transformed to a scale ranging from 0 to 10 (raw score / theoretical maximum score * 10; for example, for the subscale ‘Glucose Management’ a raw score of 12 leads to a transformed score of 12 / 15 * 10 = 8). A transformed score of ten thus represented the highest self-rating of the assessed behaviour. If ‘not required as a part of my treatment’ had been marked in an item, it was not used, and the scale score computation was adapted accordingly (by reducing the theoretical maximum score by three points). However, in case of more than half of the items of a scale missing, a scale score should not be computed.

The questionnaire was translated into English using a standardised forward and backward translation procedure, as recommended by Bradley [54]. Two independent bilingual speakers and experts in diabetes treatment performed the forward translation. The results were reviewed and matched by the developmental team. An independent bilingual speaker familiar with the field completed the backward translation. Ultimately, the back-translated and original questionnaires were matched, and the English version was finalised.

#### Summary of Diabetes Self-Care Activities Measure (SDSCA)

The SDSCA is an 11 item scale, which assesses several self-care activities by the patient’s report on the previous week. The respondent marks the number of days of the week on which the indicated behaviours were performed. The questionnaire’s first ten items are summed to a total score and pairwise averaged to five scale scores. The five scales are called ‘General Diet’ , ‘Specific Diet’ , ‘Exercise’ , ‘Blood-Glucose Testing’ , and ‘Foot-Care’ , and represent the corresponding behaviours (‘General Diet’ regards to a prescribed or generally helpful diet, whereas the items of ‘Specific Diet’ assess the consumption of ‘fruits and vegetables’ and ‘high fat foods’). The eleventh item regards smoking and assesses the average number of cigarettes smoked per day.

A review of seven studies [22] reported good consistencies (with the exception of the scale ‘Specific diet’) as well as adequate retest-reliability and criterion validity of the scales: The mean inter-item-correlation of the scale items was *r* = 0.47, the mean retest-correlation of scales was *r* = 0.40, and the mean of criterion-related correlations (estimated for ‘General Diet’ , ‘Specific Diet’ , and ‘Exercise’) was *r* = 0.23.

In this study, reliability of the SDSCA’s sum scale as determined by Cronbach’s α coefficient was 0.63. For the scales ‘General Diet’ , ‘Exercise’ , ‘Blood-Glucose Testing’ , and ‘Foot-Care’ coefficients between 0.69 and 0.88 were observed. However, the scale ‘Specific Diet’ demonstrated a strikingly low consistency according to its α coefficient of 0.15, which corresponds to the results by Toobert et al. [22].

#### Glycaemic control

Glycated haemoglobin values were used as indicator of glycaemic control. All blood samples were analysed in the German Diabetes Center’s laboratory using high performance liquid chromatography (HPLC) performed with the Bio-Rad Variant II Turbo analyser. The period between blood sampling and questionnaire assessment was usually less than one week.

### Statistical analyses

The analyses were performed using SYSTAT 10.2 (Systat Software, Point Richmond, CA, USA) and SPSS 21.0.0 (SPSS Inc., Chicago, IL, USA). Group comparisons involved One-way Analysis of Variance, Student’s t-test and Pearson’s chi-squared test. In all analyses a *P*-value of < 0.05 (two-tailed test) was considered as criterion of statistical significance.

To evaluate item characteristics, item difficulty indices (defined as percentage of agreements among all responses), inter-item-correlations and corrected item-total-correlations were computed, and the items were analysed for an increase of the scale’s reliability coefficient (Cronbach’s α) in case of item deletion. Additionally, the items’ correlations with the HbA_1c_ value were analysed. To estimate the scales’ internal consistencies, Cronbach’s α coefficients were computed. All item analyses were based on inverted item scores.

Exploratory principal component factor analysis (EFA) was used to evaluate the scale’s content structure. In this process, the varimax rotation was employed, as it usually produces explicit results which can facilitate the interpretation. Furthermore, it was assumed that the assessed self-care activities do not necessarily have to be correlated, which also suggests the orthogonal varimax rotation.

Confirmatory factor analysis (CFA) was performed using AMOS 21.0.0 to test the model defined by the EFA as well as a single factor model using the maximal likelihood estimation method. To evaluate the model fit, the Chi^2^/df ratio, comparative fit index (CFI), root mean square error of approximation (RMSEA), and the *P*-value of close fit (PCLOSE) were analysed. Adequate model fit is considered to be indicated by a Chi^2^/df ratio < 2 [55], a CFI value ≥ 0.90 [56], a RMSEA value < 0.08, and a PCLOSE > 0.05 [57].

To estimate the instrument’s validity, criterion-related correlations were analysed. The criteria were the SDSCA scales of self-care and the clinical outcomes BMI and HbA_1c_. Since DSMQ scores as well as SDSCA scores were not normally distributed, Spearman’s correlations (*ρ*) were used. Patient characteristics such as sex, age, diabetes type, diabetes duration, type of medical therapy, and number of late complications were included in the analyses to examine possible associations (in case of the dichotomous variables sex, diabetes type, and use of insulin point-biserial correlations were estimated).

Additionally, known groups validity was assessed by assorting the patients into three groups according to the HbA_1c_ value, which were then examined regarding self-care activities as assessed by the DSMQ. Patients with HbA_1c_ values up to 7.5% were classified as ‘good glycaemic control’ , patients with values between 7.6 and 8.9% were classified as ‘medium glycaemic control’, and patients with values from 9.0% as ‘poor glycaemic control’. Between-groups differences were analysed using One-way Analyses of Variance.

To evaluate the instrument’s utility for the prediction of glycaemic control, the correlations of its scales with HbA_1c_ were compared to those of the equivalent scales of the SDSCA. Differences were tested for statistical significance using Steiger’s Z test of the difference between correlated correlations, as recommended by Meng, Rosenthal & Rubin [58].

If feasible (according to the sample sizes), the explained analyses were additionally performed on the basis of the diabetes type 1 and 2 subsamples in order to test the applicability of the questionnaire in both diabetes types.

## Results

### Study 1: Development of the 16 item scale

In order to perform the item selection, 110 patients were assessed with the preliminary set of 37 items. The patients’ mean age was 51 ± 16 years, 44% were female and the mean BMI was 30 ± 7 kg/m^2^. 46% were diagnosed with type 1 diabetes and the average duration of the illness was 16 ± 10 years. The majority used an exclusive (64%) or medication-combined insulin therapy (22%), while only 13% used non-insulin medical treatments. The mean HbA_1c_ was 8.5 ± 1.8% and 53% of the patients were diagnosed with one or more late complications.

In a first step, 10 items without significant correlation with HbA_1c_ (two-sided *P* ≥ 0.05) were removed. The relevant items assessed dealing with hypoglycaemic episodes, calculation of carbohydrates, alcohol consumption, carriage of needed therapy devices, and weight control. The remaining 27 items showed correlations with the HbA_1c_ value between −0.19 and −0.43.

In a second step, two items which were found to decrease the internal consistency of this item selection were removed. For the remaining 25 items an α coefficient of 0.93 was observed.

In a third step, a principal component factor analysis was performed. The analysis identified five factors with eigenvalues higher than 1, which explained 64% of the variance. Varimax-rotated factor loadings were evaluated, and six items which did not show a loading of 0.50 or higher on any factor were removed. A renewed analysis of the remaining 19 items revealed a four factor structure, which still explained 61% of the variance.

In a fourth step, the factors were interpreted and the matching of items was rated. The factors could be easily interpreted as ‘dietary habits’ , ‘blood glucose measurement/ medication intake’ , ‘contact with health-care professionals’ , and ‘physical activity’. Regarding the associated items, there were three significant deviations: Firstly, one item which asks for overall self-care loaded primarily on ‘dietary habits’ (0.67). It was removed consequently. Secondly, one item which asks for the recording of blood glucose levels showed indeed a loading of 0.44 on the ‘blood glucose measurement/ medication intake’ factor, but it was primarily related to ‘contact with health-care professionals’ (0.61). Despite its bidimensionality and with a view to its correlation with HbA_1c_ of 0.38, it was decided to keep the item. Thirdly, one item showed substantial loadings (> 0.30) on all four factors. As this item regards overall self-care (‘my diabetes self-care is poor’), the pattern of factor loadings was rated to indicate an appropriate matching.

In the final step, the remaining 18 items were analysed for contentual redundancy. Among the items of ‘medication intake’ and ‘dietary habits’ , there were each two items of equivalent content and equal connotation. In each case, the item with the lower correlation with HbA_1c_ was removed.

According to the structure and its contents, four subscales were identified and labelled ‘Glucose Management’ (five items), ‘Dietary Control’ (four items), ‘Physical Activity’ (three items), and ‘Health-Care Use’ (three items). One additional item which addresses overall self-care (‘my diabetes self-care is poor’) is included in the ‘Sum Scale’ only (16 items).

### Study 2: Evaluation of the 16 item scale

The psychometric properties of the final 16 item version of the DSMQ were assessed in 261 patients. The SDSCA served as comparison to assess the quality of our scale. The sample characteristics are presented in Table 2. The sample was generally well matched to the first study’s sample, except that 58% of the patients were diagnosed with type 1 diabetes, which is 12% more than in the first study. However, with an average age of 52 ± 15 years, 44% female sex, a mean BMI of 30 ± 7 kg/m^2^, and a mean HbA_1c_ value of 8.6 ± 1.5% in this study, the two samples were highly comparable. Despite the slightly different proportions of diabetes types, rates of specific treatments, mean diabetes durations, and late complication statuses were highly similar (as can be seen in Table 2).

**Table 2:** Characteristics of the study sample

	**Total**	**Type 1 DM**	**Type 2 DM**	***P*-value**^**a**^
	***N *= 261**	***n *= 150 (57.5%)**	***n *= 111 (42.5%)**	
Female gender	110 (42.1%)	68 (45.3%)	42 (37.8%)	0.225
Age (years)	52.0 ± 14.9	45.8 ± 14.8	60.4 ± 10.2	<0.001
BMI (kg/m^2^)	29.7 ± 6.9	26.3 ± 4.7	34.4 ± 6.6	<0.001
Diabetes duration (years)	17.5 ± 10.4	19.0 ± 11.2	15.3 ± 8.8	0.003
Insulin therapy^b^	241 (92.3%)	150 (100%)	91 (82%)	<0.001
Exclusively insulin	186 (71.3%)	147 (98%)	39 (35.1%)	<0.001
Combined with medication^c^	55 (21.1%)	3 (2%)	52 (46.8%)	<0.001
Non-insulin medical therapy^c^	20 (7.7%)	0 (0%)	20 (18%)	<0.001
With late complication(s)^d^	132 (50.6%)	56 (37.3%)	76 (68.5%)	<0.001
Number per concerned person^d^	1.8 ± 1.1	1.4 ± 0.9	2.1 ± 1.1	<0.001
HbA_1c_ value (%)	8.6 ± 1.5	8.4 ± 1.4	8.8 ± 1.7	0.070
DSMQ ‘Sum Scale’	6.8 ± 1.7	6.9 ± 1.7	6.6 ± 1.6	0.121
Subscale ‘Glucose Management’	7.5 ± 2.3	7.5 ± 2.3	7.5 ± 2.3	0.959
Subscale ‘Dietary Control’	5.4 ± 2.4	5.2 ± 2.4	5.7 ± 2.4	0.146
Subscale ‘Physical Activity’	5.8 ± 2.7	6.5 ± 2.6	4.8 ± 2.6	<0.001
Subscale ‘Health-Care Use’	8.3 ± 1.9	8.5 ± 1.9	8.1 ± 2.0	0.085

Data are *n* (%) or M ± SD.

BMI, Body Mass Index; HbA_**1c**_, glycated haemoglobin; DSMQ, Diabetes Self-Management Questionnaire; M, mean; SD, standard deviation.

^a^ regards differences between diabetes types; Student’s t-Test or Chi^2^-Test (two-tailed test).

^b^ any type of diabetes therapy which includes the use of insulin.

^c^ oral antidiabetic agents and/or incretin mimetics.

^d^ retinopathy, neuropathy, nephropathy, diabetic foot, and/or arterial occlusive disease.

#### Item characteristics and reliability

Item analyses revealed a mean item difficulty of 46.7 (SD = 25.5). However, the indices of items 3, 4, and 7 were located in the peripheral zones of the distribution. The mean inter-item-correlation (or homogeneity) was 0.25 (SD = 0.15). The mean item-subscale-correlations were 0.56 (SD = 0.09) for ‘Glucose Management’, 0.57 (SD = 0.05) for ‘Dietary Control’, 0.59 (SD = 0.10) for ‘Physical Activity’, and 0.43 (SD = 0.01) for ‘Health-Care Use’. For the ‘Sum Scale’ a mean item-total-correlation of 0.46 (SD = 0.12) was observed, and in no case an item deletion led to an increase of the scale’s α coefficient (see Table 3). Two items (14, 15), however, showed item-total-correlations lower than 0.30. Still, those were highly correlated with their corresponding subscales. All items were negatively related to HbA_1c_ with a mean correlation of −0.23 (SD = 0.09). With the exception of the items 8 and 15, both on physical activity, all correlations with HbA_1c_ were significant. A detailed overview of the above item characteristics is displayed in Table 3.

**Table 3:** **Distribution of scores, item difficulties, scale-correlations, internal consistency in case of deletion, and correlations with HbA**_**1c **_**of the DSMQ items**

**Item**	**Distribution of item scores**	**Difficulty index**^**a**^	**Item-subscale-correlation**^**b**^	**Item-total-correlation**^**b**^	**α if item deleted**	**Correlation with HbA**_**1c**_
1	2.28 ± 0.90	78.9	0.69	0.59	0.82	−0.35‡
2	1.56 ± 0.82	53.3	0.58	0.62	0.82	−0.28‡
3	2.68 ± 0.65	94.3	0.42	0.32	0.83	−0.20†
4	2.76 ± 0.55	96.2	0.53	0.49	0.83	−0.27‡
5	1.62 ± 1.04	45.2	0.62	0.46	0.83	−0.17†
6	1.97 ± 1.12	65.9	0.49	0.47	0.83	−0.24‡
7	2.67 ± 0.63	6.5	0.44	0.35	0.83	−0.19†
8	1.36 ± 0.93	44.4	0.52	0.39	0.83	−0.09
9	1.43 ± 0.85	43.7	0.60	0.51	0.82	−0.16†
10	1.99 ± 1.07	31.4	0.60	0.55	0.82	−0.33‡
11	2.04 ± 1.01	31.4	0.70	0.41	0.83	−0.15*
12	2.31 ± 0.96	19.5	0.48	0.40	0.83	−0.30‡
13	1.91 ± 1.04	36.4	0.50	0.50	0.82	−0.30‡
14	2.15 ± 0.98	26.8	0.43	0.26	0.84	−0.20†
15	1.83 ± 1.03	38.7	0.55	0.28	0.84	−0.11
16	1.91 ± 1.01	33.7	n/a	0.69	0.81	−0.38‡

Data are M ± SD, difficulty indices, Pearson’s correlations, Cronbach’s α or Spearman’s *ρ*.

Correlations with HbA_1c_ are Spearman’s *ρ*; * *P* < 0.05; † *P* < 0.01; ‡ *P* < 0.001 (two-tailed test).

^a^ percentage of agreements among all responses.

^b^ part-whole-corrected.

Reliability analyses revealed good internal consistency of the ‘Sum Scale’ and acceptable consistencies of the subscales (except the subscale ‘Health-Care Use’ which showed a marginal consistency value). Cronbach’s α coefficients were 0.77 for ‘Glucose Management’, 0.77 for ‘Dietary Control’, 0.76 for ‘Physical Activity’, and 0.60 for ‘Health-Care Use’. For the ‘Sum Scale’ an α coefficient of 0.84 was observed.

If item and scale properties were assessed in the diabetes type subsamples separately, the analyses collectively revealed comparable results. In type 1 diabetes patients, the mean inter-item-correlation was 0.30 (SD = 0.14), the mean item-subscale-correlation was 0.58 (SD = 0.07), and the mean item-total-correlation was 0.51 (SD = 0.11). All items showed negative associations with HbA_1c_ with a mean correlation of −0.25 (SD = 0.11), and with the exception of items 8, 11, and 15 all coefficients were significant. The DSMQ subscales showed α coefficients of averagely 0.76 (SD = 0.05) and the ‘Sum Scale’s α was 0.87.

In type 2 patients, the mean inter-item-correlation was 0.20 (SD = 0.17), the mean item-subscale-correlation was 0.50 (SD = 0.12), and the mean item-total-correlation was 0.40 (SD = 0.16). All items were negatively related to the HbA_1c_ value with a mean correlation of −0.22 (SD = 0.09)_._ However, in five cases (items 8, 9, 11, 14, and 15) the correlations were insignificant. The DSMQ scales’ α coefficients were averagely 0.68 (SD = 0.12) for the four subscales and 0.80 for the ‘Sum Scale’.

#### Factorial validity

EFA suggested a four factor structure according to the Kaiser-Guttman criterion explaining 60% of variance. This result was supported by the scree test. The varimax rotation converged in 6 iterations. In view of the items’ factor loadings the factors represented the contents of ‘effective blood glucose measurement and medication intake’ (items 1, 4, 6, 10, 12), ‘dietary habits facilitating diabetes control’ (items 2, 5, 9, 13), ‘avoidance of physical exercise’ (items 8, 11, 15), and ‘avoidance of medical appointments’ (items 3, 7, 14). Item 6, which asks for the recording of blood glucose levels, again (as in the first study) revealed a bidimensional structure with its additional loading on the diet factor. The global item 16 loaded substantially (≥ 0.30) on all factors except ‘avoidance of medical appointments’. The factor loadings are presented in Table 4.

**Table 4:** Rotated factor loadings of the DSMQ items

**Items**		**Factor 1**	**Factor 2**	**Factor 3**	**Factor 4**
1	Check blood sugar levels with care and attention	**0.72**	0.34	0.00	−0.10
4	Take diabetes medication as prescribed	**0.67**	0.16	−0.02	−0.19
6	Record blood sugar levels regularly	**0.50**	0.44	0.18	−0.22
10	Do not check blood sugar levels frequently enough	**−0.76**	−0.12	0.18	0.14
12	Forget to take/ skip diabetes medication	**−0.76**	0.01	0.08	0.03
2	Choose food to easily achieve optimal blood sugar	0.32	**0.71**	−0.14	−0.11
5	Occasionally eat lots of sweets/ high-carb foods	−0.10	**−0.79**	0.02	0.02
9	Follow specialist’s dietary recommendations	0.12	**0.79**	−0.12	−0.02
13	Sometimes have real ‘food binges’	−0.10	**−0.59**	0.29	0.17
8	Do physical activity to achieve optimal sugar levels	0.13	0.31	**−0.67**	0.14
11	Avoid physical activity, although good for diabetes	−0.10	−0.12	**0.87**	0.05
15	Skip planned physical activity	0.02	−0.00	**0.82**	0.12
3	Keep recommended doctors’ appointments	0.12	0.12	−0.01	**−0.71**
7	Avoid diabetes-related doctors’ appointments	−0.15	−0.14	0.02	**0.70**
14	Should see medical practitioner(s) more often	−0.13	0.03	0.05	**0.77**
16	Diabetes self-care is poor	**−0.48**	**−0.43**	**0.35**	0.23

Extraction method: Principal component analysis. Rotation method: Varimax.

Items are shortened for ease of presentation; related factor loadings are printed in bold.

To test the observed factor structure, all items except item 16 were aggregated to four correlated factors (as suggested by the EFA) using CFA. The analysis revealed the following model fit indices: The Chi^2^/df ratio was 1.64, the CFI value was 0.96, the RMSEA value was 0.05, and the PCLOSE was 0.50. These results indicate a very appropriate fit of the four factor model. To evaluate the feasibility of integrating all items to a total scale, an additional single factor model (all 16 items aggregated on one factor) was tested. The analysis revealed a Chi^2^/df ratio of 1.74, a CFI value of 0.95, a RMSEA value was 0.053, and a PCLOSE of 0.34, which indicated an adequate fit of this model, too.

#### Known-groups validity

The comparison of patient groups with ‘good glycaemic control’ (HbA_1c_ ≤ 7.5%), ‘medium glycaemic control’ (HbA_1c_ 7.6 – 8.9%), and ’poor glycaemic control’ (HbA_1c_ ≥ 9.0%) revealed significant differences regarding both the DSMQ sum scores as well as the subscale scores. All results are shown in Table 5.

**Table 5:** **Comparison of the DSMQ self-care activities in patients with HbA**_**1c **_**≤ 7.5%, from 7.6 to 8.9%, and ≥ 9.0%**

**DSMQ**	**HbA**_**1c **_**≤ 7.5%**	**Sign.**^**a**^	**HbA**_**1c **_**7.6–8.9%**	**Sign.**^**b**^	**HbA**_**1c **_**≥ 9.0%**	**Sign.**^**c**^	**ANOVA**
**self-care activities**	**(*n *= 67)**		**(*n *= 106)**		**(*n *= 88)**		***P*-value**
Glucose Management	8.7 ± 1.6	*	7.8 ± 2.0	‡	6.4 ± 2.5	‡	<0.001
Dietary Control	6.4 ± 2.1	ns	5.6 ± 2.3	†	4.5 ± 2.4	‡	<0.001
Physical Activity	6.6 ± 2.7	*	5.5 ± 2.7	ns	5.5 ± 2.6	*	0.021
Health-Care Use	8.7 ± 1.6	ns	8.5 ± 1.9	ns	7.9 ± 2.1	*	0.013
Sum Scale	7.7 ± 1.2	†	6.9 ± 1.4	‡	5.9 ± 1.8	‡	<0.001

Data are M ± SD. Tests were One-way ANOVA and Scheffé Test for post-hoc group comparisons. Scheffé Test significance is expressed: * *P* < 0.05; † *P* < 0.01; ‡ *P* < 0.001; ns, not significant.

DSMQ, Diabetes Self-Management Questionnaire; HbA_**1c**_, glycated haemoglobin; ANOVA, Analysis of Variance.

^a^ regards comparison between the first and second group.

^b^ regards comparison between the second and third group.

^c^ regards comparison between the third and first group.

According to these results, patients with ‘good glycaemic control’ reported significantly more ‘Glucose Management’, ‘Dietary Control’, ‘Physical Activity’, and ‘Health-Care Use’ than those with ‘poor control’. Correspondingly, in this group the mean ‘Sum Scale’ score was significantly higher.

Compared to the ‘medium glycaemic control’ group, patients with ‘good control’ reported significantly more ‘Glucose Management’ and ‘Physical Activity’. Furthermore, they had a higher ‘Sum Scale’ score than those with ‘medium control’. However, significant differences regarding ‘Dietary Control’ and ‘Health-Care Use’ were not observed.

Patients with ‘medium glycaemic control’ , on the other hand, reported significantly more ‘Glucose Management’ and ‘Dietary Control’ than those with ‘poor control’ , and they also had a higher ‘Sum Scale’ score. However, no significant differences were observed regarding ‘Physical Activity’ and ‘Health-Care Use’.

#### Convergent validity

The DSMQ’s associations with external criteria (patient characteristics, BMI, SDSCA scales, and HbA_1c_ value) as observed in the total sample as well as the diabetes type specific subsamples are presented in Table 6.

**Table 6:** **Correlations between the DSMQ scales and patient characteristics, SDSCA scales, and HbA**_**1c **_**as assessed in the total sample and type 1 (in parenthesis) and type 2 (in square brackets) diabetes patient subgroups**

	**DSMQ**				
	**Glucose Management**	**Dietary Control**	**Physical Activity**	**Health-Care Use**	**Sum Scale**
Female gender	0.03	−0.03	−0.00	0.09	0.01
	(−0.07)	(−0.00)	(0.06)	(0.11)	(0.00)
	[0.18]	[−0.06]	[−0.15]	[0.05]	[0.02]
Age	0.32‡	0.40‡	−0.05	0.12*	0.28‡
	(0.44‡)	(0.41‡)	(0.16*)	(0.27†)	(0.44‡)
	[0.27†]	[0.36‡]	[0.05]	[0.15]	[0.31†]
BMI	0.00	−0.07	**−0.41**‡	−0.08	−0.22‡
	(0.03)	(−0.11)	(−**0.30**‡)	(0.01)	(−0.15)
	[−0.08]	[−0.23†]	[−**0.27**†]	[−0.06]	[−0.25†]
Diabetes type 1	−0.00	−0.09	0.31‡	0.11	0.10
	(−)	(−)	(−)	(−)	(−)
	[−]	[−]	[−]	[−]	[−]
Diabetes duration	0.10	0.02	0.09	0.15*	0.13*
	(0.12)	(−0.02)	(0.07)	(0.12)	(0.08)
	[0.07]	[0.13]	[0.01]	[0.18]	[0.18]
Insulin therapy^a^	0.09	0.02	0.03	0.05	0.08
	(−0.09)	(−0.05)	(−0.04)	(0.13)	(−0.05)
	[0.17]	[0.10]	[−0.10]	[−0.00]	[0.10]
Number of late complications^b^	0.05	0.24‡	−0.14*	0.01	0.08
	(0.09)	(0.07)	(−0.06)	(0.12)	(0.07)
	[0.02]	[0.37‡]	[0.03]	[−0.03]	[0.22†]
SDSCA Blood-Glucose Testing	**0.57**‡	0.28‡	0.24‡	0.26‡	0.51‡
	(**0.58**‡)	(0.37‡)	(0.29‡)	(0.28‡)	(0.56‡)
	[**0.58**‡]	[0.25†]	[0.09]	[0.19*]	[0.44‡]
SDSCA General Diet	0.29‡	**0.52**‡	0.18†	0.13*	0.44‡
	(0.38‡)	(**0.55**‡)	(0.29‡)	(0.26†)	(0.54‡)
	[0.20*]	[**0.44**‡]	[0.17]	[−0.03]	[0.36‡]
SDSCA Specific Diet	0.08	**0.28**‡	0.19†	0.08	0.24‡
	(0.01)	(**0.28**‡)	(0.20*)	(0.22†)	(0.26†)
	[0.16]	[**0.26**†]	[0.23†]	[−0.09]	[0.23]
SDSCA Exercise	0.05	0.17†	**0.58**‡	0.06	0.30‡
	(0.09)	(0.22†)	(**0.60**‡)	(0.10)	(0.35‡)
	[−0.01]	[0.12]	[**0.53**‡]	[−0.02]	[0.18]
SDSCA Foot-Care	0.25‡	0.35‡	0.03	0.10	0.29‡
	(0.23)	(0.25†)	(0.19*)	(0.16)	(0.31‡)
	[0.30†]	[0.44‡]	[0.05]	[0.10]	[0.39‡]
SDSCA Smoking^c^	−0.22‡	−0.15*	−0.09	−0.19†	−0.20†
	(−0.31‡)	(−0.23†)	(−0.22†)	(−0.29†)	(−0.32‡)
	[−0.06]	[0.04]	[−0.03]	[−0.09]	[−0.02]
SDSCA Sum scale	0.37‡	0.54‡	0.39‡	0.17†	**0.57**‡
	(0.35‡)	(0.55†)	(0.51‡)	(0.29†)	(**0.62**‡)
	[0.38‡]]	[0.49‡]	[0.34‡]	[0.02]	[**0.51**‡]
HbA_1c_ value	**−0.39**‡	**−0.30**‡	−0.15*	**−0.22**‡	**−0.40**‡
	(−**0.44**‡)	(−**0.31**‡)	(−**0.12**)	(−0.20*)	(−**0.39**‡)
	[−**0.33**‡]	[−**0.33**‡]	[−**0.11**]	[−0.21*]	[−**0.38**‡]

Coefficients are Spearman’s *ρ* or point-biserial correlation (regards the dichotomous variables female sex, diabetes type 1, and insulin therapy); * *P* < 0.05; † *P* < 0.01; ‡ *P* < 0.001 (two-tailed test).

Coefficients which represent type 1 patients (n = 150) are presented in parenthesis; coefficients which represent type 2 patients (n = 111) are presented in square brackets; coefficients which are indicative of convergent validity are printed in bold.

DSMQ, Diabetes Self-Management Questionnaire; BMI, Body Mass Index; SDSCA; Summary of Diabetes Self-Care Activities Measure; HbA_**1c**_, glycated haemoglobin.

^a^ any type of diabetes therapy which includes the use of insulin.

^b^ retinopathy, neuropathy, nephropathy, diabetic foot, and/or arterial occlusive disease.

^c^ average number of cigarettes smoked per day.

The examination of the DSMQ’s correlations in the total sample of 261 patients revealed the following results: The subscale ‘Glucose Management’ was highly correlated with the equivalent SDSCA scale ‘Blood-Glucose Testing’ (*ρ* = 0.57) and the HbA_1c_ value (*ρ* = −0.39). The subscale ‘Dietary Control’ was highly correlated with the equivalent SDSCA scale ‘General Diet’ (*ρ* = 0.52) and substantially with ‘Specific Diet’ (*ρ* = 0.28). Furthermore, it showed a substantial negative correlation with HbA_1c_ (*ρ* = −0.30). The subscale ‘Physical Activity’ was highly correlated with the equivalent SDSCA scale ‘Exercise’ (*ρ* = 0.58). Its correlation with the HbA_1c_ value was −0.15 and there was also a high negative correlation with the BMI (*ρ* = −0.41). Regarding the subscale ‘Health-Care Use’ there is no equivalent scale of the SDSCA. Nevertheless, it was significantly correlated with the SDSCA scales ‘General Diet’ (*ρ* = 0.13), ‘Blood-Glucose Testing’ (*ρ* = 0.26), ‘Foot Care’ (*ρ* = 0.10), and ‘Smoking’ (*ρ* = −0.19), and showed a substantial negative correlation with the HbA_1c_ value of −0.22. Finally, the DSMQ ‘Sum Scale’ showed substantial to high correlations between 0.20 and 0.51 with all SDSCA scales and was highly correlated with the SDSCA’s total score with 0.57. Its negative correlation with the HbA_1c_ value was high (*ρ* = −0.40).

If convergent correlations were assessed separately by diabetes type, the analyses of both subsamples revealed results which were highly comparable to those presented above. All DSMQ subscales as well as the ‘Sum Scale’ still showed significant correlations of equivalent sizes with their relevant convergent criteria (see Table 6). However, one exception was observed regarding the subscale ‘Physical Activity’: Although it showed slight correlations with HbA_1c_ in both types of diabetes patients, none of those reached statistical significance.

#### DSMQ vs. SDSCA: Associations with HbA_1c_

The comparison between the DSMQ scales and their equivalent SDSCA scales regarding the correlations with HbA_1c_ (and for the physical activity scales with BMI) revealed the following results:

As in the case with the DSMQ subscale ‘Glucose Management’ , the SDSCA’s equivalent scale ‘Blood-Glucose Testing’ was significantly correlated with the HbA_1c_ value (*ρ* = −0.22, *P* < 0.001). However, the correlation between ‘Glucose Management’ and HbA_1c_ (*ρ* = −0.39, *P* < 0.001) was significantly higher (*Z* = −3.07, *P* < 0.01).

While the SDSCA scale ‘Specific Diet’ was not correlated with HbA_1c_ (*ρ* = −0.02, *P* = 0.746), the scale ‘General Diet’ was (*ρ* = −0.13, *P* = 0.042). However, the DSMQ subscale ‘Dietary Control’ showed a higher correlation (*ρ* = −0.30, *P* < 0.001), and again the difference was significant (*Z* = −2.84, *P* < 0.01).

In contrast to the DSMQ subscale ‘Physical Activity’ (*ρ* = −0.15, *P* = 0.013), the equivalent SDSCA scale ‘Exercise’ was not correlated with HbA_1c_ (*ρ* = 0.07, *P* = 0.239), and the difference between correlations was significant (*Z* = −3.96, *P* < 0.001). Additionally, ‘Physical Activity’ showed a higher correlation with the BMI (*ρ* = −0.41, *P* < 0.001) than the SDSCA scale ‘Exercise’ (*ρ* = −0.18, *P* = 0.004), and this difference again was significant (*Z* = −4.33, *P* < 0.001).

In contrast to the DSMQ ‘Sum Scale’ , which showed a notable correlation with the HbA_1c_ value of −0.40 (*P* < 0.001), the SDSCA’s total score was not significantly correlated with HbA_1c_ (*ρ* = −0.10, *P* = 0.123). This difference was highly significant (*Z* = −5.39, *P* < 0.001).

When these correlational analyses were performed separately by diabetes type, the results were in total clearly consistent with the ones described above. In both diabetes types the DSMQ scales ‘Glucose Management’, ‘Dietary Control’ and ‘Sum Scale’ showed significantly higher correlations with HbA_1c_ than their equivalent SDSCA scales (all *P* < 0.05). However, neither the DSMQ subscale ‘Physical Activity’ nor its equivalent ‘Exercise’ were significantly correlated with HbA_1c_ in the subsamples (all *P* > 0.10). Therefore, the finding of a higher association between the DSMQ subscale and HbA_1c_ – as observed in the total sample – could not be replicated. Nevertheless, comparably to the total sample evaluation, ‘Physical Activity’ showed higher correlations with the BMI than the SDSCA scale ‘Exercise’ in both subsamples. However, only in the type 2 patients sample reached this difference statistical significance (*Z* = −2.20, *P* < 0.05).

## Discussion and conclusions

The purpose of this investigation was to describe the development of the DSMQ (study 1) and evaluate its psychometric properties (study 2). The questionnaire was developed on a broad theoretical and empirical basis, and its evaluation indicates very good psychometric properties with adequate item characteristics, satisfactory reliability, and good validity.

According to the generally satisfactory item properties and good item validity coefficients regarding HbA_1c_ the overall item selection appears very satisfying. Since the items assess a number of different aspects of self-care, the total scale is rather heterogeneous, which is reflected by the mean inter-item-correlation of 0.25. Against this background and with a view to the rather low number of items on each content, the internal consistency can be appraised as good (based on the standard by Nunnally and Bernstein [59]). For a polydimensional construct a higher alpha coefficient might even be unfavourable, for it suggests high item redundancy in the scale, as pointed out by Streiner [60]. The slightly lower item-total-correlations in two cases should be interpreted with a view to this aspect as well. The additional analyses of the subsamples revealed slightly better item properties and consistency in type 1 patients which can be partly attributed to the difference in sample size. In sum, all coefficients were in the acceptable range and suggest general applicability.

The EFA revealed a simple structure of four factors with high loadings of all items thereon. The factors were well interpretable and their contents clearly confirmed the designed scales. One discrepancy could be seen in item 6, which belongs to the subscale ‘Glucose Management’ but showed an additional loading on the dietary factor. But apart from that, the overall content structure is remarkably clear and indicates a good factorial validity. The EFA revealed a very good fit of the suggested four factor model, which also confirms the designed scales. Additionally, a single factor model was found to fit the data as well, which suggests the feasibility of the integration of all item scores to the ‘Sum Scale’.

The criterion-related correlations between the DSMQ scales and the SDSCA scales indicate a good convergence between parallel measures suggesting validity. The finding that all parallel scales show a strong convergence (> 0.5) has to be stressed particularly because the questionnaires employ markedly different time frames (one week in the SDSCA in contrast to eight weeks in the DSMQ) which might actually discount those correlations. Additionally, the throughout significant correlations with the objective outcome measure HbA_1c_ confirm the assumption of validity and, moreover, prove the questionnaire’s high utility for the intended scientific but also clinical purposes. The additional analyses of convergent correlations by diabetes type revealed comparably strong associations with external criteria in both type 1 and type 2 diabetes and provide good evidence of the DSMQ’s general applicability.

The known groups analysis showed significant differences between patient groups with ‘good’ , ‘medium’ , and ‘poor’ glycaemic control, which provides evidence of the questionnaire’s ability to discriminate between patients’ behaviours. According to these results, higher sum scores as well as subscale scores of the DSMQ allow to infer better self-care activities in view of glycaemic control.

Notably, the DSMQ and SDSCA are equivalent in the way that both questionnaires assess self-care activities, which in most cases are clearly related, as reflected by the correlations between the parallel scales. However, in spite of this commonality, self-care as assessed by the DSMQ is more strongly associated with glycated haemoglobin, which can be explained by the differently conceptualized functions [19; p. 367 et seq.]. Furthermore, the DSMQ’s timeframe focusses the relevant behaviours of the previous two months which apparently allows a more reliable assessment of self-care and a better prediction of the glycaemic outcome.

In the course of the item selection only self-care activities which showed relevant associations with glycaemic control were kept. For this reason, several specific self-care activities which may be of interest in regards of diabetes care are not covered by the DSMQ. However, the precise choice of contents is essential to ensure the questionnaire’s focus on self-care predictive of glycaemic control. It sum, it can be stated that the DSMQ’s development, particularly with a view to its specific objectives, appears clearly successful.

The main limitation of the studies is based on the composition of the samples. Both samples were drawn from in-patients at a tertiary referral centre for diabetes, where patients are usually hospitalized because of relevant problems of diabetes treatment and glycaemic control (reflected by the average HbA_1c_ values of 8.5 and 8.6% in the samples), and showed a relatively long average diabetes duration and a high prevalence of late complications. Therefore, the study participants cannot be rated as representative of the general diabetic population, which limits the generalizability of results [61]. Furthermore, the majority of patients was treated with insulin, whereas only a small percentage used non-insulin medical treatments. Thus, the pattern of correlations between the DSMQ scales and HbA_1c_ might differ when assessed in patients not treated with insulin or antidiabetic medication (for example, dietary aspects and physical activity then might have a larger impact on glycaemic control). For this reason, the properties demonstrated here should primarily be attributed to the questionnaire’s use in insulin-treated patients, for the present. However, with the exception of ‘medication intake’ (which is obviously related to medical regimens) all contents assessed by the DSMQ can be literarily related to glycaemic control regardless of the type of treatment [34,40,45-47,51]. Finally, although a wide spectrum of adult ages was covered in study 2 (from 18 to 86 years), data on the questionnaire’s use in youths or children are not available yet, suggesting further research in this regard.

Due to the generally short length of stay at the GDCM, the investigation was carried out cross-sectionally. Since no retest was performed, there is no information on the instrument’s stability or sensitivity to change. Furthermore, information on the questionnaire’s relations to common behavioural and psychological variables associated with diabetes care is currently still limited. In these regards additional analyses are needed. Nevertheless, the present results may be judged as promising.

The strengths of this investigation, on the other hand, lie in the theoretical and empirical basis of the questionnaire contents on recent results from self-care research, which facilitates the integration of our findings and supports face validity. The questionnaire development was performed through a highly formal process of item and test analysis (study 1), and its initial validation (study 2) was based on a very appropriate sample size. Furthermore a high accuracy of HbA_1c_ analysis was achieved (due to standardised analysis in a central laboratory), and the coincidence of blood sampling and psychometric assessment as well as the standardized data assessment ensure the internal validity of results.

Regarding its associations with HbA_1c,_ the DSMQ showed significant superiority to the German version of the SDSCA. It could be argued that the SDSCA’s lower correlations were the consequence of translation problems. However, already the original English version’s initial evaluation could not relate any of its scales with glycated haemoglobin [19], and this result is supported by studies from several countries, which did not find significant associations of the SDSCA scales with HbA_1c_ either [62-65]. Against this background, the present findings appear conclusive, suggesting that the DSMQ’s superiority may be attributed to the differences of construct assessment between the instruments.

In sum, in this initial study the DSMQ demonstrated very good psychometric properties. The questionnaire presents itself as an efficient instrument which provides reliable and valid information on diabetes self-care, and assesses four well-defined specific self-care activities associated with glycaemic control. It was designed especially to enable scientific studies of psychosocial barriers to self-care and glycaemic control. However, since good metabolic control can be regarded as the most important goal of diabetes treatment, the questionnaire appears also valuable for the clinical use as a screener or as diagnostic instrument to assess barriers of glycaemic control in individuals. Thus, the DSMQ should benefit future research and also be of value in clinical settings.

## Abbreviations

ANOVA: Analysis of variance; BMI: Body mass index; CFA: Confirmatory factor analysis; CFI: Comparative fit index; DSMQ: Diabetes Self-management questionnaire; EFA: Exploratory factor analysis; GDCM: German diabetes center mergentheim; HbA1c: Glycated haemoglobin; M: Mean; PCLOSE: P-value of close fit; RMSEA: Root mean square error of approximation; SD: Standard deviation; SDSCA: Summary of diabetes self-care activities measure; SMBG: Self-monitoring of blood glucose.

## Competing interests

The authors declare that they have no competing interests.

## Authors’ contributions

AS developed the questionnaire, designed/ carried out the study, analysed the data and drafted the manuscript. AG contributed to study design and article revision. NH contributed to study design, questionnaire translation and article revision. BK contributed to the article revision. JH contributed to the questionnaire translation and article revision. TH contributed to study design and article revision. All authors read and approved the final manuscript.

## References

[B1] Spellman CW Achieving glycemic control: cornerstone in the treatment of patients with multiple metabolic risk factors J Am Osteopath Assoc 2009 109 Suppl 5 8 13 19451256

[B2] Stettler C Allemann S Jüni P Cull CA Holman RR Egger M Krähenbühl S Diem P Glycemic control and macrovascular disease in types 1 and 2 diabetes mellitus: Meta-analysis of randomized trials Am Heart J 2006 152 27 38 10.1016/j.ahj.2005.09.015 16824829

[B3] Akalin S Berntorp K Ceriello A Das AK Kilpatrick ES Koblik T Munichoodappa CS Pan CY Rosenthall W Shestakova M Wolnik B Woo V Yang WY Yilmaz MT Global Task Force on Glycaemic Control Intensive glucose therapy and clinical implications of recent data: a consensus statement from the Global Task Force on Glycaemic Control Int J Clin Pract 2009 63 1421 1425 10.1111/j.1742-1241.2009.02165.x 19769698

[B4] Johnson SB Methodological issues in diabetes research. Measuring adherence Diabetes Care 1992 15 1658 1867 10.2337/diacare.15.11.1658 1468298

[B5] Albisser AM Harris RI Albisser JB Sperlich M The impact of initiatives in education, self-management training, and computer-assisted self-care on outcomes in diabetes disease management Diabetes Technol Ther 2001 3 571 579 10.1089/15209150152811199 11911169

[B6] Williams GC McGregor HA Zeldman A Freedman ZR Deci EL Testing a Self-Determination Theory Process Model for Promoting Glycemic Control Through Diabetes Self-Management Health Psychol 2004 23 58 66 14756604 10.1037/0278-6133.23.1.58

[B7] Piette JD Richardson C Valenstein M Addressing the needs of patients with multiple chronic illnesses: the case of diabetes and depression Am J Manag Care 2004 10 152 162 15005508

[B8] Peyrot M McMurry JF Jr Kruger DF A biopsychosocial model of glycemic control in diabetes: stress, coping and regimen adherence J Health Soc Behav 1999 40 141 158 10.2307/2676370 10467761

[B9] Gonzalez JS Peyrot M McCarl LA Collins EM Serpa L Mimiaga MJ Safren SA Depression and diabetes treatment nonadherence: a meta-analysis Diabetes Care 2008 31 2398 2403 10.2337/dc08-1341 19033420 PMC2584202

[B10] Gonzalez JS Delahanty LM Safren SA Meigs JB Grant RW Differentiating symptoms of depression from diabetes-specific distress: relationships with self-care in type 2 diabetes Diabetologia 2008 51 1822 1825 10.1007/s00125-008-1113-x 18690422 PMC2678064

[B11] Aikens JE Perkins DW Lipton B Piette JD Longitudinal analysis of depressive symptoms and glycemic control in type 2 diabetes Diabetes Care 2009 32 1177 1181 10.2337/dc09-0071 19389814 PMC2699713

[B12] Fisher L Glasgow RE Strycker LA The relationship between diabetes distress and clinical depression with glycemic control among patients with type 2 diabetes Diabetes Care 2010 33 1034 1036 10.2337/dc09-2175 20150291 PMC2858170

[B13] Fisher L Mullan JT Arean P Glasgow RE Hessler D Masharani U Diabetes distress but not clinical depression or depressive symptoms is associated with glycemic control in both cross-sectional and longitudinal analyses Diabetes Care 2010 33 23 28 10.2337/dc09-1238 19837786 PMC2797978

[B14] Lustman PJ Anderson RJ Freedland KE de Groot M Carney RM Clouse RE Depression and poor glycemic control: a meta-analytic review of the literature Diabetes Care 2000 23 934 942 10.2337/diacare.23.7.934 10895843

[B15] Pibernik-Okanovic M Grgurevic M Begic D Szabo S Metelko Z Interaction of depressive symptoms and diabetes-related distress with glycaemic control in Type 2 diabetic patients Diabet Med 2008 25 1252 1254 19046209 10.1111/j.1464-5491.2008.02553.x

[B16] Baron RM Kenny DA The moderator-mediator variable distinction in social psychological research: conceptual, strategic, and statistical considerations J Pers Soc Psychol 1986 51 1173 1182 3806354 10.1037//0022-3514.51.6.1173

[B17] Lustman PJ Clouse RE Ciechanowski PS Hirsch IB Freedland KE Depression-related hyperglycemia in type 1 diabetes: a mediational approach Psychosom Med 2005 67 195 199 10.1097/01.psy.0000155670.88919.ad 15784783

[B18] Glasgow RE Chur BC Social-environmental factors in diabetes: Barriers to diabetes self-care Handbook of Psychology and Diabetes: a guide to psychological measurement in diabetes research and practice 1994 Switzerland: Harwood Academic 335 349

[B19] Toobert DJ Glasgow RE Chur BC Assessing diabetes self-management: the summary of diabetes self-care activities questionnaire Handbook of Psychology and Diabetes: a guide to psychological measurement in diabetes research and practice 1994 Switzerland: Harwood Academic 351 375

[B20] Wang RH Lin LY Cheng CP Hsu MT Kao CC The psychometric testing of the diabetes health promotion self-care scale J Nurs Res 2012 20 122 130 10.1097/jnr.0b013e318254eb47 22592107

[B21] Lee NP Fisher WP Jr Evaluation of the Diabetes Self-Care Scale J Appl Meas 2005 6 366 381 16192661

[B22] Toobert DJ Hampson SE Glasgow RE The summary of diabetes self-care activities measure: results from 7 studies and a revised scale Diabetes Care 2000 23 943 950 10.2337/diacare.23.7.943 10895844

[B23] Eigenmann CA Colagiuri R Skinner TC Trevena L Are current psychometric tools suitable for measuring outcomes of diabetes education? Diabet Med 2009 26 425 436 10.1111/j.1464-5491.2009.02697.x 19388974

[B24] Bastos F Severo M Lopes C Psychometric analysis of diabetes self-care scale (translated and adapted to Portuguese) Acta Med Port 2007 20 11 20 17624279

[B25] Choi EJ Nam M Kim SH Park CG Toobert DJ Yoo JS Chu SH Psychometric properties of a Korean version of the summary of diabetes self-care activities measure Int J Nurs Stud 2011 48 333 337 10.1016/j.ijnurstu.2010.08.007 20950807

[B26] Michels MJ Coral MH Sakae TM Damas TB Furlanetto LM Questionnaire of Diabetes Self-Care Activities: translation, cross-cultural adaptation and evaluation of psychometric properties Arq Bras Endocrinol Metabol 2010 54 644 651 10.1590/S0004-27302010000700009 21085770

[B27] Vincent D McEwen MM Pasvogel A The validity and reliability of a Spanish version of the summary of diabetes self-care activities questionnaire Nurs Res 2008 57 101 106 10.1097/01.NNR.0000313484.18670.ab 18347481

[B28] Kav S Akman A Dogan N Tarakci Z Bulut Y Hanoglu Z Turkish validity and reliability of the summary of diabetes self-care activities measure for patients with type 2 diabetes mellitus J Clin Nurs 2010 19 2933 2935 10.1111/j.1365-2702.2010.03329.x 20846234

[B29] Morris AD Boyle DI McMahon AD Greene SA MacDonald TM Newton RW Adherence to insulin treatment, glycaemic control, and ketoacidosis in insulin-dependent diabetes mellitus. The DARTS/MEMO Collaboration. Diabetes Audit and Research in Tayside Scotland. Medicines Monitoring Unit Lancet 1997 350 1505 1510 10.1016/S0140-6736(97)06234-X 9388398

[B30] Donnelly LA Morris AD Evans JM DARTS/MEMO collaboration Adherence to insulin and its association with glycaemic control in patients with type 2 diabetes QJM 2007 100 345 350 10.1093/qjmed/hcm031 17504861

[B31] Lawrence DB Ragucci KR Long LB Parris BS Helfer LA Relationship of oral antihyperglycemic (sulfonylurea or metformin) medication adherence and hemoglobin A1c goal attainment for HMO patients enrolled in a diabetes disease management program J Manag Care Pharm 2006 12 466 471 16925454 10.18553/jmcp.2006.12.6.466 PMC10437554

[B32] Krapek K King K Warren SS George KG Caputo DA Mihelich K Holst EM Nichol MB Shi SG Livengood KB Walden S Lubowski TJ Medication adherence and associated hemoglobin A1c in type 2 diabetes Ann Pharmacother 2004 38 1357 1362 10.1345/aph.1D612 15238621

[B33] Cohen HW Shmukler C Ullman R Rivera CM Walker EA Measurements of medication adherence in diabetic patients with poorly controlled HbA(1c) Diabet Med 2010 27 210 216 10.1111/j.1464-5491.2009.02898.x 20546266 PMC4626013

[B34] Thomas D Elliott EJ Low glycaemic index, or low glycaemic load, diets for diabetes mellitus Cochrane Database Syst Rev 2009 1 CD006296 10.1002/14651858.CD006296.pub2 PMC6486008 19160276

[B35] Thomas DE Elliott EJ The use of low-glycaemic index diets in diabetes control Br J Nutr 2010 104 797 802 10.1017/S0007114510001534 20420752

[B36] Wikblad K Montin K Wibell L Metabolic control, residual insulin secretion and self-care behaviours in a defined group of patients with type 1 diabetes Ups J Med Sci 1991 96 47 61 10.3109/03009739109179258 1897062

[B37] Schütt M Kern W Krause U Busch P Dapp A Grziwotz R Mayer I Rosenbauer J Wagner C Zimmermann A Kerner W Holl RW DPV Initiative Is the frequency of self-monitoring of blood glucose related to long-term metabolic control? Multicenter analysis including 24,500 patients from 191 centers in Germany and Austria Exp Clin Endocrinol Diabetes 2006 114 384 388 10.1055/s-2006-924152 16915542

[B38] Malanda UL Welschen LM Riphagen II Dekker JM Nijpels G Bot SD Self-monitoring of blood glucose in patients with type 2 diabetes mellitus who are not using insulin Cochrane Database Syst Rev 2012 1 CD005060 10.1002/14651858.CD005060.pub3 PMC11984800 22258959

[B39] Poolsup N Suksomboon N Rattanasookchit S Meta-analysis of the benefits of self-monitoring of blood glucose on glycemic control in type 2 diabetes patients: an update Diabetes Technol Ther 2009 11 775 784 10.1089/dia.2009.0091 20001678

[B40] Allemann S Houriet C Diem P Stettler C Self-monitoring of blood glucose in non-insulin treated patients with type 2 diabetes: a systematic review and meta-analysis Curr Med Res Opin 2009 25 2903 2913 10.1185/03007990903364665 19827909

[B41] St John A Davis WA Price CP Davis TM The value of self-monitoring of blood glucose: a review of recent evidence J Diabetes Complications 2010 24 129 141 10.1016/j.jdiacomp.2009.01.002 19230717

[B42] Hirsch IB Bode BW Childs BP Close KL Fisher WA Gavin JR Ginsberg BH Raine CH Verderese CA Self-Monitoring of Blood Glucose (SMBG) in insulin- and non-insulin-using adults with diabetes: consensus recommendations for improving SMBG accuracy, utilization, and research Diabetes Technol Ther 2008 10 419 439 10.1089/dia.2008.0104 18937550

[B43] Polonsky WH Fisher L Self-monitoring of blood glucose in noninsulin-using type 2 diabetic patients: right answer, but wrong question: self-monitoring of blood glucose can be clinically valuable for noninsulin users Diabetes Care 2013 36 179 182 10.2337/dc12-0731 23264290 PMC3526239

[B44] Speight J Browne JL Furler J Challenging evidence and assumptions: is there a role for self-monitoring of blood glucose in people with type 2 diabetes not using insulin? Curr Med Res Opin 2013 29 161 168 10.1185/03007995.2012.761957 23259703

[B45] Boulé NG Haddad E Kenny GP Wells GA Sigal RJ Effects of exercise on glycemic control and body mass in type 2 diabetes mellitus: a meta-analysis of controlled clinical trials JAMA 2001 286 1218 1227 10.1001/jama.286.10.1218 11559268

[B46] Thomas DE Elliott EJ Naughton GA Exercise for type 2 diabetes mellitus Cochrane Database Syst Rev 2006 3 CD002968 10.1002/14651858.CD002968.pub2 PMC8989410 16855995

[B47] Tonoli C Heyman E Roelands B Buyse L Cheung SS Berthoin S Meeusen R Effects of different types of acute and chronic (training) exercise on glycaemic control in type 1 diabetes mellitus: a meta-analysis Sports Med 2012 42 1059 1080 23134339 10.1007/BF03262312

[B48] Parchman ML Pugh JA Noël PH Larme AC Continuity of care, self-management behaviors, and glucose control in patients with type 2 diabetes Med Care 2002 40 137 144 10.1097/00005650-200202000-00008 11802086

[B49] Sidorenkov G Voorham J Haaijer-Ruskamp FM de Zeeuw D Denig P Association Between Performance Measures and Glycemic Control Among Patients With Diabetes in a Community-wide Primary Care Cohort Med Care 2013 51 172 179 10.1097/MLR.0b013e318277eaf5 23222526

[B50] Schectman JM Schorling JB Voss JD Appointment adherence and disparities in outcomes among patients with diabetes J Gen Intern Med 2008 23 1685 1687 10.1007/s11606-008-0747-1 18661189 PMC2533370

[B51] Karter AJ Parker MM Moffet HH Ahem AT Ferrara A Liu JY Selby JV Missed appointments and poor glycemic control: An opportunity to identify high-risk diabetic patients Med Care 2004 42 110 115 10.1097/01.mlr.0000109023.64650.73 14734947

[B52] Nathan DM Turgeon H Regan S Relationship between glycated haemoglobin levels and mean glucose levels over time Diabetologia 2007 50 2239 2244 10.1007/s00125-007-0803-0 17851648 PMC8752566

[B53] Nathan DM Kuenen J Borg R Zheng H Schoenfeld D Heine RJ the A1c-Derived Average Glucose (ADAG) Study Group Translating the A1C assay into estimated average glucose values Diabetes Care 2008 31 1473 1478 10.2337/dc08-0545 18540046 PMC2742903

[B54] Bradley C Chur BC Translation of questionnaires for use in different languages and cultures Handbook of Psychology and Diabetes: a guide to psychological measurement in diabetes research and practice 1994 Switzerland: Harwood Academic Publishers 43 55

[B55] Marsh HW Hocevar D Application of confirmatory factor analysis to the study of self-concept: First- and higher order factor models and their invariance across groups Psychol Bull 1985 97 562 582

[B56] Bentler PM Comparative fit indexes in structural models Psychol Bull 1990 107 238 246 2320703 10.1037/0033-2909.107.2.238

[B57] Browne MW Cudeck R Bollen KA, Long JS Alternative ways of assessing model fit Testing Structural Equation Models 1993 Beverly Hills, CA: Sage 136 162

[B58] Meng X Rosenthal R Rubin DB Comparing correlated correlation coefficients Psychol Bull 1992 111 172 175

[B59] Nunnally J Bernstein L Psychometric theory 1994 3 New York: McGraw-Hill Higher, INC

[B60] Streiner DL Starting at the beginning: an introduction to coefficient alpha and internal consistency J Pers Assess 2003 80 99 103 10.1207/S15327752JPA8001_18 12584072

[B61] Bornhöft G Maxion-Bergemann S Wolf U Kienle GS Michalsen A Vollmar HC Gilbertson S Matthiessen PF Checklist for the qualitative evaluation of clinical studies with particular focus on external validity and model validity BMC Med Res Methodol 2006 6 56 10.1186/1471-2288-6-56 17156475 PMC1764896

[B62] Song M Ratcliffe SJ Tkacs NC Riegel B Self-care and health outcomes of diabetes mellitus Clin Nurs Res 2012 21 309 326 10.1177/1054773811422604 21926278

[B63] Amsberg S Anderbro T Wredling R Lisspers J Lins PE Adamson U Johansson UB Experience from a behavioural medicine intervention among poorly controlled adult type 1 diabetes patients Diabetes Res Clin Pract 2009 84 76 83 10.1016/j.diabres.2008.12.011 19181414

[B64] Primožič S Tavčar R Avbelj M Dernovšek MZ Oblak MR Specific cognitive abilities are associated with diabetes self-management behavior among patients with type 2 diabetes Diabetes Res Clin Pract 2012 95 48 54 10.1016/j.diabres.2011.09.004 21963107

[B65] Tan SL Juliana S Sakinah H Dietary compliance and its association with glycemic control among poorly controlled type 2 diabetic outpatients in Hospital Universiti Sains Malaysia Malays J Nutr 2011 17 287 299 22655451

